# [18F]FDG-labelled stem cell PET imaging in different route of administrations and multiple animal species

**DOI:** 10.1038/s41598-021-90383-4

**Published:** 2021-05-25

**Authors:** Naoko Nose, Suguru Nogami, Kazuhiro Koshino, Xinyu Chen, Rudolf A. Werner, Soki Kashima, Steven P. Rowe, Constantin Lapa, Kazuki Fukuchi, Takahiro Higuchi

**Affiliations:** 1 Graduate School of Medicine, Dentistry and Pharmaceutical Sciences, Okayama University, Okayama, Japan; 2 Department of Medical Physics and Engineering, Division of Health Sciences, Osaka University Graduate School of Medicine, Suita, Japan; 3 Department of Systems and Informatics, Hokkaido Information University, Ebetsu, Japan; 4 Comprehensive Heart Failure Center and Department of Nuclear Medicine, University Hospital Würzburg, Oberdürrbacher Strasse 6, 97080 Würzburg, Germany; 5 Nuclear Medicine, Medical Faculty, University of Augsburg, Augsburg, Germany; 6 The Russell H. Morgan Department of Radiology and Radiological Science, Johns Hopkins University School of Medicine, Baltimore, MD USA; 7 Department of Urology, Akita University Graduate School of Medicine, Akita, Japan

**Keywords:** Stem cells, Molecular medicine, Biomarkers, Stem-cell research

## Abstract

Stem cell therapy holds great promise for tissue regeneration and cancer treatment, although its efficacy is still inconclusive and requires further understanding and optimization of the procedures. Non-invasive cell tracking can provide an important opportunity to monitor in vivo cell distribution in living subjects. Here, using a combination of positron emission tomography (PET) and in vitro 2-deoxy-2-[18F]fluoro-D-glucose ([18F]FDG) direct cell labelling, the feasibility of engrafted stem cell monitoring was tested in multiple animal species. Human mesenchymal stem cells (MSCs) were incubated with phosphate-buffered saline containing [18F]FDG for in vitro cell radiolabelling. The pre-labelled MSCs were administrated via peripheral vein in a mouse (n = 1), rats (n = 4), rabbits (n = 4) and non-human primates (n = 3), via carotid artery in rats (n = 4) and non-human primates (n = 3), and via intra-myocardial injection in rats (n = 5). PET imaging was started 10 min after cell administration using a dedicated small animal PET system for a mouse and rats. A clinical PET system was used for the imaging of rabbits and non-human primates. After MSC administration via peripheral vein, PET imaging revealed intense radiotracer signal from the lung in all tested animal species including mouse, rat, rabbit, and non-human primate, suggesting administrated MSCs were trapped in the lung tissue. Furthermore, the distribution of the PET signal significantly differed based on the route of cell administration. Administration via carotid artery showed the highest activity in the head, and intra-myocardial injection increased signal from the heart. In vitro [18F]FDG MSC pre-labelling for PET imaging is feasible and allows non-invasive visualization of initial cell distribution after different routes of cell administration in multiple animal models. Those results highlight the potential use of that imaging approach for the understanding and optimization of stem cell therapy in translational research.

## Introduction

In recent decades, stem cell therapy has gained broad interest due to its potential for tissue regenerative medicine^1,2^. Multiple preclinical and clinical studies have shown promising positive effects of grafted stem cells on the repair and regeneration of various tissues, such as in stroke and myocardial infarction^3^. Additionally, stem cells have been shown to migrate into tumours, making them potential vehicles for anticancer therapy^4–6^.

In the clinical practice of cell therapy, one of the major technical issues is how to efficiently deliver the stem cells into the target tissues and the route of administration needs to be taken into special consideration^7,8^. Intravenous cell administration is the easiest and relatively non-invasive route, allowing for repeated injection. However, such systemic administration may require a larger amount of total cell dose and needs sufficient and long-distance cell migration into the target area. Selective cell administration via an arterial blood vessel, perfusing the target tissue, is minimally-invasive and would be expected to improve the efficacy of cell delivery. At the same time, it carries risks of embolism and of poor delivery to hypoperfused areas. Finally, intra-tissue direct cell injection could further improve the number of delivered cells, even in hypo-perfused regions, while there may be spillover to nontarget organs and loss of cells due to mechanical alterations. Nevertheless, direct injection is an invasive procedure which may limit clinical application depending on the location of the target tissue. There is also a need for a better understanding of the dynamics of administered cells in vivo, as the efficacy of delivery and cell survival may vary depending on the types of stem cell and the target tissue/organ^9^.

Non-invasive imaging is a relevant tool for monitoring and optimizing the procedure of cell therapy in living subjects^10–12^. Radionuclide molecular imaging is a routinely available technology for the visualization of key molecular and cellular alterations in patients and animal models. Positron emission tomography (PET) can serve as a highly sensitive and quantitative approach for visualizing those molecular and cellular events. Methods utilizing PET technology to monitor stem cells have been proposed^13,14^. For instance, Patrick *et al*. recently demonstrated the usefulness of [89Zr]Zr-oxine labelling and PET imaging for evaluating the biodistribution of novel, highly innovative cell therapies^15^. Promising results have been also achieved with 3’-deoxy-3’-[18F]fluorothymidine^16^. In vitro pre-labelling with the glucose analog 2-deoxy-2-[18F]fluoro-D-glucose ([18F]FDG), followed by cell administration and PET imaging, however, is one of the simplest and well established approaches for monitoring stem cell distribution in living organisms^17,18^, in particular as [18F]FDG is widely available at every PET facility with access to a cyclotron^19^. We tested the feasibility to assess stem cell distribution in multiple animal species after different routes of administration using PET imaging.

## Material and methods

All reagents were commercial products and used without further purification unless otherwise indicated. Animal studies were approved by the local institutional animal ethics committee (approved number 18019, National Cardiovascular Research Center, Osaka, Japan) and performed according to the Guide for the Care and Use of Laboratory Animals published by the U.S. National Institutes of Health (NIH publication 85-23, revised 1996)^20^ and ARRIVE guidelines (https://arriveguidelines.org).

### Radiopharmaceuticals

[18F]FDG was supplied from Nihon Medi-Physics Co., Ltd., Tokyo, Japan. Radiochemical purity was greater than 95% for radiolabelled compounds.

### Cell preparation

Human MSCs from bone marrow were purchased from PromoCell GmbH (Heidelberg, Germany) and were cultured in Mesenchymal Stem Cell Growth Medium 2 (MSCGM2, PromoCell GmbH). Media was changed after 24 h to select for adherent cells and subsequently every 3 days until 80% confluence was reached. MSCs were cultivated and passaged as needed, never exceeding passage 5 for use in subsequent experiments.

### [18F]FDG cell uptake and washout assay

[18F]FDG uptake study with MSCs was performed as previously reported^21^ with a minor modification. In brief, cells (1.0–3.0 × 10^6^ cells/well in 6-well plate) were washed with Dulbecco's phosphate-buffered saline (DPBS, Nacalai Tesque Inc., Kyoto, Japan). DPBS containing 4.5–5.5 MBq/mL [18F]FDG was added to each well. After 60 min incubation (37 °C, 5% CO_2_), the medium was removed and [18F]FDG uptake into the cells was immediately terminated by applying 2 ml of ice-cold DPBS. The cells were washed twice with 2 ml of ice-cold DPBS and then lysed with 0.1 M NaOH for 60 min for protein and radioactivity measurements. The protein of each cell lysate was measured using a BCA Protein Assay Kit (Thermo Scientific™ Pierce™ BCA Protein Assay Kit, Thermo Fisher Scientific K.K., Tokyo, Japan) and iMark™ Microplate Absorbance Reader (Bio-Rad Laboratories, Inc., CA, USA). The radioactivity of each lysate was measured by a γ-counter (1480 WIZARD™ 3, PerkinElmer, Inc., MA, USA). For uptake kinetics assessment (n = 3), cells were incubated with [18F]FDG for different incubation times (5 min, 15 min, 30 min, 60 min and 120 min), with and without glucose in DPBS (2.0 mg/mL). For the assessment of radiotracer washout from the cells, after the 60 min cell labelling, the cells were kept in the DPBS (37 °C, 5% CO_2_) not containing [18F]FDG for different time periods (60 min, 180 min and 360 min), then washed two times and the counts remaining in the cells were measured.

### Cell preparation for in vivo animal study

For PET imaging, MSCs were incubated with DPBS containing 4.5–10.0 MBq/mL [18F]FDG for 60 min (37 °C, 5% CO_2_) and resuspended in 1.0 mL of 37 °C pre-warmed DPBS after being washed three times with DPBS.

### Animals

All animals fasted overnight with free access to water up to 1 h before anaesthesia. For in vivo experiments, male C57BL/6 mouse (n = 1, weighing 21 g, Charles River Laboratories JAPAN, Inc., Kanagawa, Japan), male Wistar rats (n = 13, 9–21 weeks, weighing 150–250 g, Japan SLC, Inc. Shizuoka, Japan), and male New Zealand white rabbits (3 y/o, weighing 3.8–4.1 kg, Japan SLC, Inc.) were used. General anesthesia was induced by using 5% isoflurane (ISOFLURANE Inhalation Solution; Pfizer Japan Inc., Tokyo, Japan) and was maintained during the experiment with 2% isoflurane. For experiments with non-human primates (NHPs) (5 males and 1 female rhesus macaques, 3.5–4.5 y/o, weighing 3.3–5.3 kg, Primate Research Institute Kyoto University, Aichi, Japan) were used. Induction of anaesthesia in NHPs was conducted by intramuscular injection of ketamine (1.5 mg/kg) and xylazine (0.6 mg/kg) to allow preparation and handling of the animals. After a tracheal cannula was inserted, 1.5% sevoflurane (SEVOFLURANE Inhalation Solution; Pfizer Japan, Inc.) vaporized with 100% oxygen was inhaled and the tidal volume and respiratory rate of the ventilator were monitored and kept in the normal range throughout the imaging sessions with an anaesthesia workstation (Apollo®, Drägerwerk AG & Co. KGaA. Lübeck, Germany). During the PET imaging session, animals were placed on the scanner bed in a prone position for mouse, rats, and rabbits and a supine position for NHPs and safely attached to keep the position during the scan. The animals were covered with blankets or laying on the heating bed to maintain body temperature.

### Cell administration in animal studies

**Figure 1:**
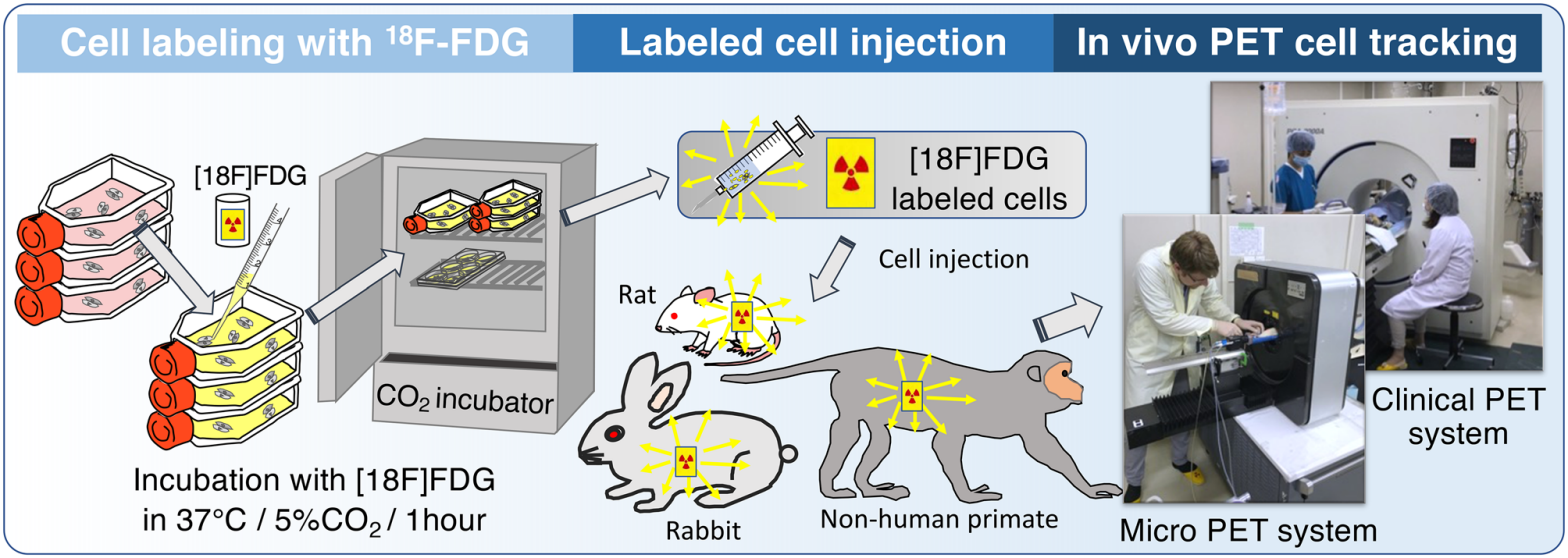
Schematic presentation of the imaging experiment procedure of cell radiolabelling, cell injection, and PET imaging.

In vivo studies are conducted using a mouse, rats, rabbits and NHPs with different route of MSC administration, and scanned at 10 min after the cell administration. Intravenous administration of MSCs were performed via tail vein in the mouse (n = 1, 8.5 × 10^4^ cells, 0.1 mL) and rats (n = 4, 4.6–19.0 × 10^5^ cells, 1.0 mL), via ear vein in the rabbits (n = 4, 1.3–2.5 × 10^7^ cells, 1.0 mL), and via arm vein in the NHPs (n = 3, 1.2–2.2 × 10^7^ cells, 1.0 mL). Administration via carotid artery (common) was performed in rats (n = 4, 4.9–16.3 × 10^5^ cells, 1.0 mL) and NHPs (n = 3, 1.2–2.2 × 10^7^ cells, 1.0 mL). Intra-myocardial injection was performed in rats (n = 5, 1.3–1.6 × 10^6^ cells, 0.25 ml) using 27G needles under thoracotomy (Fig. 1).

### PET imaging

PET scans of mouse and rats were obtained using a dedicated small-animal PET system (microPET FOCUS 120, Siemens Healthcare K.K., Tokyo, Japan), while a clinical PCA-2000A positron scanner (Toshiba Medical Systems Corporation, Tochigi, Japan) was used for the scans of rabbits and NHPs. PET imaging was started 10 min after the injection of [18F]FDG–labelled MSCs. Whole-body PET acquisitions were obtained (total 30 min for one bed position for a mouse, total 90 min for three bed positions for rats, 30 min for 3 bed positions for rabbits, and 40 min for 4 bed positions for NHPs). The data were sorted into 3-dimensional sinograms, which were then reconstructed with a Fourier transformation to produce dynamic images using a 2-dimensional ordered-subset expectation maximization (OSEM) algorithm. All images were corrected for fluorine-18 decay, random and dead time; correction for attenuation was not performed for mouse and rats, but attenuation correction was performed for rabbits and NHPs^22^. The obtained PET images were analysed with the public domain tool AMIDE imaging software (A Medical Imaging Data Examiner, version 1.01, http://amide.sourceforge.net/).

Following the PET imaging session of rats, the hearts, lungs, and brains were harvested for analysis of tissue counts with a γ-counter (1480 WIZARD™ 3). Following decay correction of tissue counts, percent injected dose (%ID) = 100 × (radioactivity in organ)/[(activity injected)] in each organ was calculated.

### Statistics

Results are given as mean ± SD. The two-tailed paired Student’s t-test was used to compare differences between dependent groups and the two-tailed independent Student’s t-test between independent groups. Multiple group comparisons were performed using analysis of variance (ANOVA) followed by Dunnett’s multiple comparison test. A value of *p* less than 0.05 was considered statistically significant.

### Ethical standards

All applicable international, national and/or institutional guidelines for the care and use of animals were followed. All experimental procedures using animals were approved by the Animal Ethics Committee of the National Cerebral and Cardiovascular Center Research Institute, Osaka, Japan.

## Results

### [18F]FDG uptake and retention

**Figure 2:**
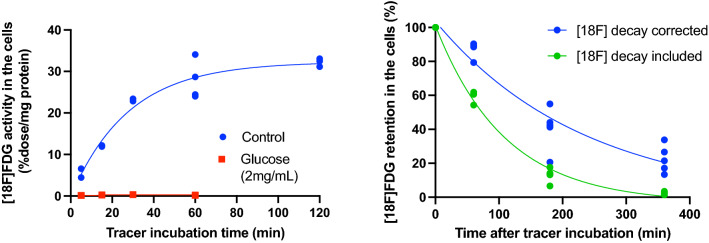
In vitro [18F]FDG uptake kinetics in MSCs (left) and radiotracer washout from the cells (right). Radiotracer activity in the cells was plateaued at 60–120 min incubation (physiological fluorine-18 decay correction). Radiotracer signal remained in the cells at a level of approximately 80–90% after 60 min, while decreased by nearly 50% after 180 min.

[18F]FDG uptake in the MSCs was completely inhibited by adding glucose (2.0 mg/mL) in the buffer media (Fig. 2). The radiotracer activity (without decay correction) increased in a time dependent manner up to 60 min and plateaued at 120 min (Fig. 2), suggesting that optimal incubation time to achieve high fluorine-18 counts from the cells is approximately 60 min. [18F]FDG washout from the cells was observed when kept in the DPBS (Fig. 2). 80–90% of fluorine-18 radioactivity remained in the MSCs after 60 min, while more than 50% of the fluorine-18 signal was washed out from the cells into the media after 180–360 min.

### PET imaging of [18F]FDG labelled MSCs after peripheral vein administration in different animals

**Figure 3:**
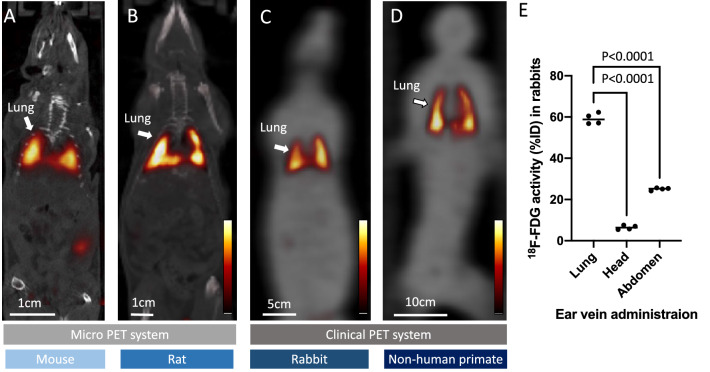
Representative whole body PET images after [18F]FDG labelled MSCs administration via peripheral vein in a mouse (**A**), rat (**B**), rabbit (**C**), and non-human primate (**D**). Images of the mouse and rat are fused with CT (gray scale) and rabbit and non-human primate are fused with transmission scan (gray scale). High radiotracer signal is seen in the lung of all tested animal species. Quantification of [18F]FDG distribution (%ID) in the lung, head, and abdomen area in rabbits (**E**).

PET imaging in a mouse, rats, rabbits, and NHPs was successfully conducted after administration of [18F]FDG labelled MSCs. In all animal species, intense radiotracer signal was detected in the bilateral lung (Fig. 3), and slightly increased activity was seen in the urinary tract system. Percentage of [18F]FDG uptake (%ID) in rabbits estimated by the PET images were 59.2 ± 2.6, 6.4 ± 1.0* and 25.1 ± 0.6* for the lung, head, and abdomen area, respectively (*vs lung, *p* < 0.0001).

### Different routes of cell administrations in rats and NHPs

**Figure 4:**
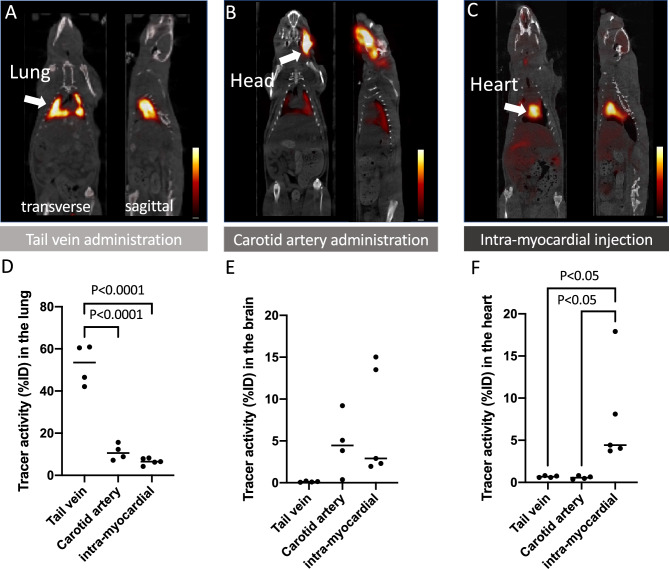
Representative PET images of rats with cell administration via tail vein (**A**), common carotid artery (**B**), and intra-myocardial injection (**C**). High radiotracer activity is seen in the lung, head, and heart in the group of animals with tail vein administration, carotid artery injection, and intra-myocardial injection, respectively. Results of tissue counting of the lung (**D**), brain (**E**), and heart (**F**) with different routes of cell administration are shown in the graphs.

**Figure 5:**
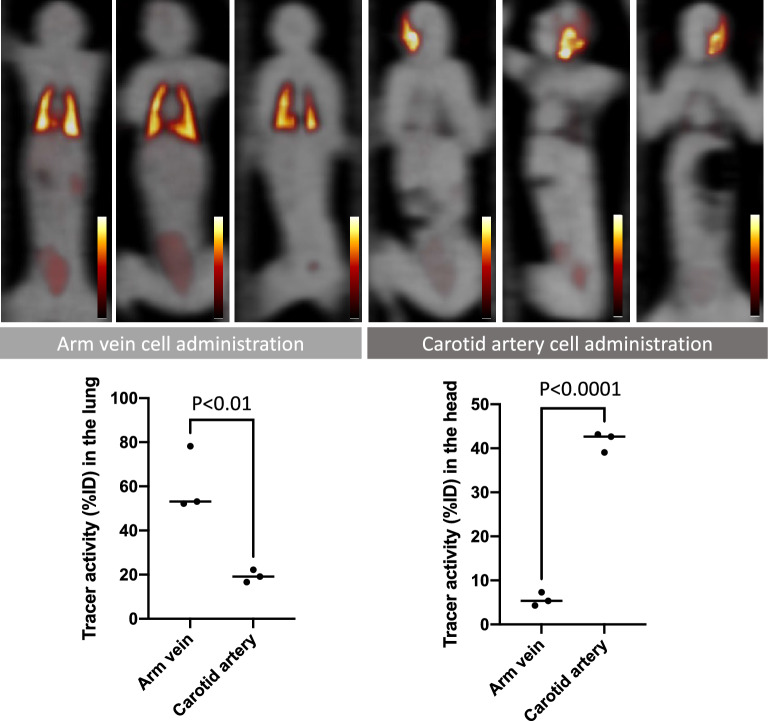
All whole-body PET coronal images of non-human primates (n = 6) after administration of [18F]FDG labeled MSCs via arm vein (n = 3, upper left) and common carotid artery (n = 3, upper right). PET images are fused with transmission scan images (gray scale). High activity in the bilateral lung is seen in the animals with cell injection via arm vein, while high activity in the head is seen in the animals injected via carotid artery. Quantitative PET signals in the lung (lower left) and the head area (lower right) with different cell administration routes are shown.

After common carotid artery cell administration and intra-myocardial cell injection in rats, completely different radiotracer distribution patterns were seen in PET images (Fig. 4). Most of the radiotracer signal remained in the head area after carotid artery administration, while a strong signal remained in the heart after intra-myocardial injection. Minimal radiotracer activity in the lungs was detected after injection through either route, unlike peripheral vein administration which led to predominant lung signal. The results of the rat experiments were consistent with the results obtained after administration to NHPs via common carotid artery (Fig. 5). High radiotracer signal at the head area (side of tracer injection) was observed, while almost no lung activity was detected in PET images of all three NHPs that received the cells via carotid artery injection.

## Discussion

In this study, we successfully visualized initial distribution of administrated MSCs in multiple animal species using a combination of highly sensitive PET imaging and simple in vitro cell pre-labelling with the glucose analogue [18F]FDG. Two different PET scanners, a high-resolution, small-animal and a large-bore-size clinical PET system, were employed to accommodate the differences in animal sizes from mouse to NHP. A typical small animal PET system has a gantry bore diameter of ~ 10–20 cm and available for small rodents such as mouse and rat^23^, while a standard clinical PET system has a gantry bore opening ~ 60–80 cm which easily accommodates NHPs^24^. To see the delicate anatomical structures in small animals, 1–2 mm of spatial resolution are achieved in small-animal scanner systems, while typically 3–4 mm of special resolution can be achieved in a clinical PET system^24,25^.

Several different techniques that utilize radionuclides for visualization of transplanted cells have been proposed that utilize radionuclides^26,27^. There two methods that have been explored most extensively are direct pre-labeling and the reporter gene approach. As shown in the current study, direct pre-labeling of the cells prior to the administration is a straightforward manner in which to visualize the delivery of the administrated cells. Multiple different radionuclides have been tested, including commercially available [111In]In-oxine kits for clinical leukocyte inflammatory imaging. For the visualization of indium-111 radioactivity signal, a gamma camera system must be used, often in single-photon emission computed tomography (SPECT) mode. Although the higher accessibility is an advantage of the SPECT technology, PET has intrinsic advantages including better temporal and spatial resolution, higher sensitivity for detection of the radiotracer, and established algorithms for attenuation correction as well as absolute quantification of distribution^28^. The radiotracer used to label the cells in this study was [18F]FDG, which is an analogue of glucose and the most widely used PET agent in the clinical arena, making it easily obtainable^17,18^. However, potential limitations of direct labeling cell imaging are the decay of the radioactivity and radiotracer leakage from the cells. In our in vitro washout assay, almost 50% of the radioactivity had diffused out of the cells at 180 min, and the radioisotope fluorine-18 decay half-life is 109.77 min. Therefore, [18F]FDG labeled cell imaging may be limited to no later than 60 min after cell injection, making this method most suitable for the evaluation of initial cell distribution and the early phase of cell engraftment.

To overcome those limitations, reporter gene cell imaging has been introduced^29–31^. In this approach, the donor cells need to be genetically modified to stably express a reporter protein that selectively takes up a specific radiotracer into the cells. Several pairs of reporter protein and reporter probe have been developed and tested in animal models, such as herpes simplex type 1 thymidine kinase (HSV1-sr39tk) as a reporter and 9-(4-[18F]fluoro-3-hydroxymethylbutyl) guanine as a probe (31), and sodium/iodine cotransporter as a symporter and iodine-123/124/131 or technetium-99m as a probe^29,30^. A key feature of reporter cell imaging is the possibility of monitoring cells over time. If the cells keep expressing the reporter protein after transplantation, repeated systemic administration of the radiotracer allows for longitudinal imaging of cell localization^30^. Although the reporter gene approach has attractive features for cell imaging, several hurdles still need to be overcome including standardization of efficient and safe cell genetic labeling technology, as well as high physiological background tracer activity and low cell detection sensitivity as compared to direct cell labeling methods.

In this PET study, clear radiotracer signal in the bilateral lung fields were detected after intravenous human MSCs administration in all tested animal species, most likely indicating donor cell entrapment. The size of human MSCs is 15–50 μm in diameter with an average of 26.5 μm^32^, whereas the average pulmonary capillary diameter is ~ 12 µm in human and mouse, i.e. much smaller than the size of MSCs^33^. Additionally, cell adhesion abilities are also suggested as a factor for the pulmonary cell trapping mechanism in capillaries^34^. Different tracer distribution in rats and NHPs after MSCs administration via common carotid artery as compared to intravenous administration could also be explained by cell entrapment at the peripheral capillary. Moreover, we could also observe high radiotracer accumulation in the head after carotid artery administration (Fig. 4B,E). In animals after focal brain injury, which have been injected with MSC into the carotid artery, MSCs were capable of homing to the perivascular space^35^, thereby showing no migration to deeper brain areas. The latter compartment is crucially involved in the replacement of injured pericytes, which facilitates restoration of central nervous system function^36^ after brain damage and is also tightly linked to MSC^37^, potentially explaining why radiotracer signal also remained in the head area after carotid artery administration in our study. Moreover, in patients afflicted with heart failure, intramyocardial injection of MSC of individuals undergoing a left ventricular assist device implant did not lead to an increased rate of weaning from device support relative to patients without MSC injection into the myocardium^38^. This was partially explained by an increased level of inflammation around the surgical intervention, potentially leading to increased death of MSC, e.g. by paracrine signaling^38^. Of note, in our study enrolling healthy animals, increased radiotracer accumulation was observed after injection of MSC into the myocardium (Fig. 4C,F). Therefore, future studies should evaluate the potential of this administration route in animals after cardiac damage, e.g. post-myocardial infarction, in particular as PET can then reveal the retention capacities of the MSC in-vivo prior to any MSC-based therapeutic intervention.

The results of the present study obtained in different species may lay the foundation for future translational studies, e.g. to track the optimal number of transplanted cells or to assess cell viability and retention to increase efficacy of MSC-based treatments. Then, preclinical stem cell monitoring studies mimicking clinical situations such as animal model of diseases and different types of donor cells are warranted. These considerations are further fueled by the fact that clinical trials e.g. on cerebral infarction and chronic ischemic heart disease and stem cell therapy have yielded mixed results^39,40^. Beyond cardiovascular disease, recent studies also demonstrated that transplanted MSCs are mainly trapped in the lung, e.g. in animal models of pulmonary emphysema mimicking cigarette smokers^41,42^. This is in contradistinction to the brain, as MSC can cross the blood brain barrier^43^. Therefore, PET imaging of [18F]FDG-labeled stem cells may reveal retention capacities in the target organ (e.g. in the lung), but can also assess the off-target response of transplanted MSC (e.g. in the brain), ultimately identifying the optimal individual which will most likely benefit from treatment with almost no or negligible side effects^44–46^.

## Conclusions

Using a combination of dedicated small animal PET and clinical PET systems, [18F]FDG pre-labelled cells were visualized after different routes of cell administration in multiple animal models. Those results highlight the potential use of PET as a research platform for the understanding of in vivo cell kinetics and optimization of procedures for stem cell therapy.
